# Supplementary material for: Integrated analysis of the aqueous humor microbiome and lens capsule transcriptome in high myopia cataract: a pilot study
Source: Front Med (Lausanne). 2026 Jun 16;13:1845205. doi: 10.3389/fmed.2026.1845205 (PMC13314463; doi:10.3389/fmed.2026.1845205)
Supplement: Supplementary file 3 [file Data_Sheet_1.zip › 3.Beta_Diversity/NMDS/C372089/NMDS_euclidean_2D.pdf]

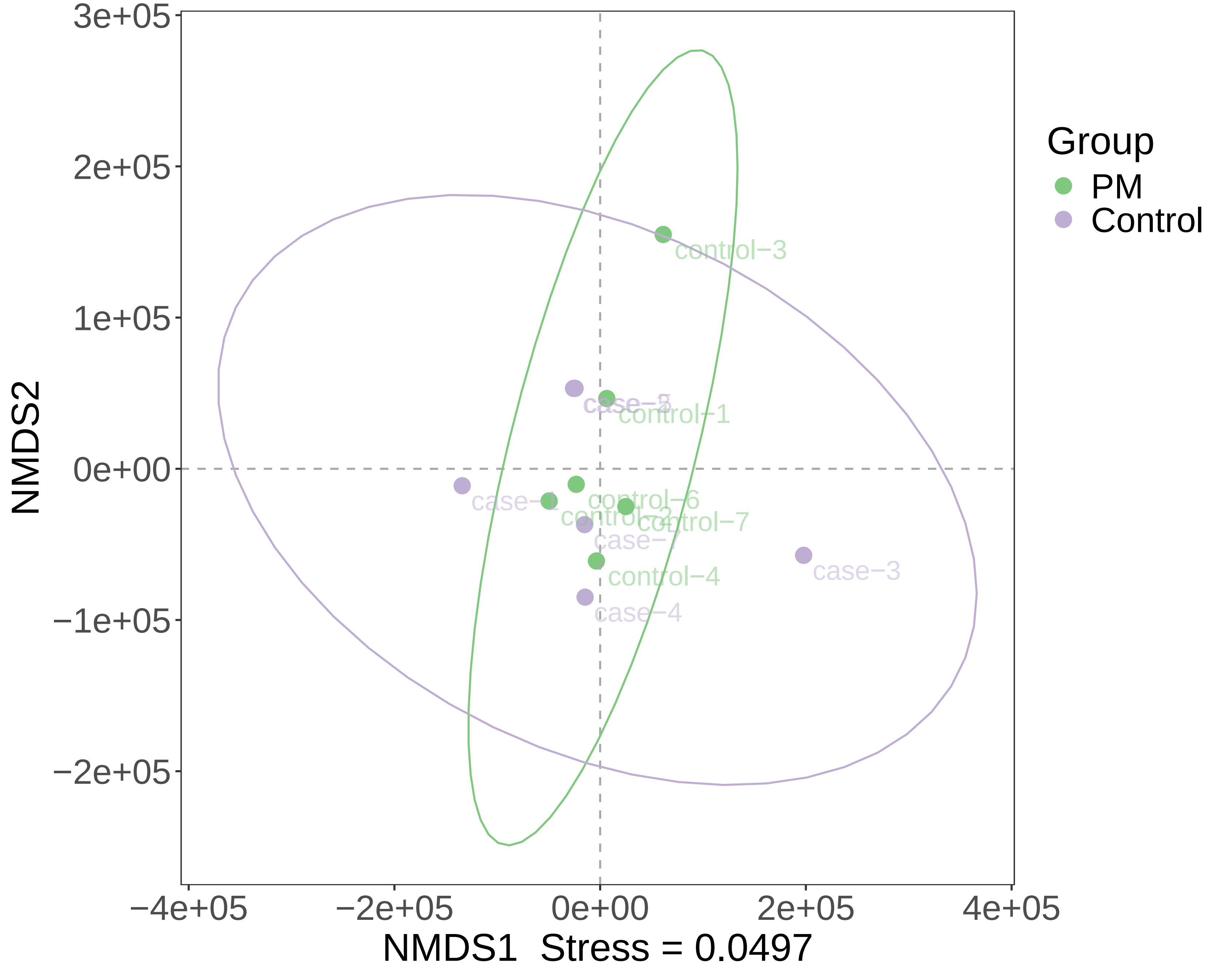

3e+05
2e+05
1e+05
0e+00
−1e+05
−2e+05
−4e+05
−2e+05
0e+00
2e+05
4e+05
NMDS2
NMDS1 Stress = 0.0497
Group
PM
Control
control−3
case−5
case−2
control−1
case−1
control−6
control−2
control−7
case−7
case−3
control−4
case−4
